# Pain Expressions and Inhibitory Control in Patients With Fibromyalgia: Behavioral and Neural Correlates

**DOI:** 10.3389/fnbeh.2018.00323

**Published:** 2019-01-08

**Authors:** Marina Pidal-Miranda, A. J. González-Villar, M. T. Carrillo-de-la-Peña

**Affiliations:** ^1^Department of Clinical Psychology and Psychobiology, University of Santiago de Compostela, Santiago de Compostela, Spain; ^2^Psychological Neuroscience Lab, Research Center in Psychology, School of Psychology, University of Minho, Braga, Portugal

**Keywords:** brain electrical activity, chronic pain, cognitive dysfunction, emotion, attention, pain, inhibitory control

## Abstract

Fibromyalgia (FM) is a generalized chronic pain condition associated with a variety of symptoms, including altered cognitive and emotional processing. It has been proposed that FM patients show a preferential allocation of attention to information related to the symptoms of the disease, particularly to pain cues. However, the existing literature does not provide conclusive evidence on the presence of this attentional bias, and its effect on cognitive functions such as inhibitory control. To clarify this issue, we recorded the electroencephalographic activity of 31 women diagnosed with FM and 28 healthy women, while performing an emotional Go/NoGo task with micro-videos of pain, happy, and neutral facial expressions. We analyzed behavioral data, performed EEG time-frequency analyses, and obtained the event-related potentials (ERPs) N2 and P3 components in NoGo trials. A series of self-reports was also administered to evaluate catastrophic thinking and the main symptoms of fibromyalgia. Pain expressions were associated with longer reaction times and more errors, as well as with higher theta and delta power, and P3 amplitude to NoGo stimuli. Thus, behavioral and psychophysiological data suggest that increased attention to pain expressions impairs the performance of an inhibitory task, although this effect was similar in FM patients and healthy controls. N2 amplitude was modulated by type of facial expression (larger to pain faces), but only for the control group. This finding suggests that the presentation of pain faces might represent a smaller conflict for the patients, more used to encounter pain stimuli. No main group effects were found significant for N2 or P3 amplitudes, nor for time-frequency data. Using stimuli with greater ecological validity than in previous studies, we could not confirm a greater effect of attentional bias toward negative stimuli over inhibitory performance in patients with FM. Studying these effects allow us to better understand the mechanisms that maintain pain and develop intervention strategies to modify them.

## Introduction

Fibromyalgia (FM) is a chronic pain condition of unknown etiology, characterized by the presence of generalized musculoskeletal pain, in addition to fatigue, poor sleep quality, anxiety, depression, and altered cognitive and emotional processing. It has been proposed that FM patients show a preferential allocation of attention to information related to the symptoms of the disease, particularly to pain cues. This idea has been discussed as hypervigilance and, more recently, as attentional bias (AB) (Crombez et al., 2013).

Attention toward nociceptive stimulation is believed to modulate the painful experience. Some studies have shown that focusing attention on noxious stimuli made them be perceived as more intense and more unpleasant than directing attention elsewhere (Garcia-Larrea and Jackson, 2016). Even observing empathically other people's pain can enhance our own intensity reports to painful stimuli (de Wied and Verbaten, 2001; Godinho et al., 2008). Therefore, the investigation of AB to pain-related information may help to understand the causal mechanisms of pain maintenance as well as the development of anxiety and depression associated to pain (Aldrich et al., 2000; Crombez et al., 2012).

Although patients with chronic pain are often thought to be characterized by hypervigilance toward pain-related information (Pearce and Morley, 1989), this assumption is controversial. Many studies have failed to replicate this result, either reporting no evidence of such bias (Pincus et al., 1998; Asmundson et al., 2005), or finding similar biases in patients, and healthy controls (Crombez et al., 2000; Snider et al., 2000; Beck et al., 2001; Andersson and Haldrup, 2003). In their 2013 review, Crombez et al. concluded that there is a bias toward pain-related words or images in patients with chronic pain conditions such as fibromyalgia. They added, however, that the effect is small, and that AB toward signals of pain was present in healthy participants as well. Therefore, it is not a robust phenomenon, nor is it easy to identify, generate or replicate (Crombez et al., 2013).

Several factors may explain the inconsistent pattern of evidence concerning AB in patients with chronic pain. Among them, the lack of ecological validity of stimuli (usually pain-related words),—that may not represent the patients' real concerns or may not be relevant to the population studied—, and the exposure time of stimuli have been underscored as critical variables (Crombez et al., 2013). In spite of the greatest potential impact of using images, their inclusion in studies investigating AB is rather recent and limited. Also, most of the studies have only analyzed behavioral indices (that is, reaction times or hit rates), which may not be sensitive enough to the effect of AB.

To overcome some of the above issues, in this study we presented dynamic faces with different expressions (pain, neutral, and happiness) using 500 ms micro-videos, and proposed a different strategy to assess the effect of AB. Our objective was to investigate how attention toward pain-related information affects response inhibition, one of the executive functions altered in fibromyalgia (Correa et al., 2011). Several authors argue that the neural circuits of inhibitory control and pain may overlap, and thus that the brain regions engaged in response inhibition might be hypoactivated in chronic pain patients (Glass et al., 2011; Schmidt-Wilcke et al., 2014). To clarify this, we recorded electrophysiological activity and behavioral data during the performance of a Go/NoGo emotional task from patients with FM and healthy controls. Due to the dynamic nature of stimuli, EEG was analyzed by time-frequency decomposition. Theta and delta activity increases have been observed during NoGo response inhibition (Harper et al., 2014), although the effect of the presence of pain on them has not been explored to date. We also analyzed the N2 and P3 components of the event-related potentials (ERPs), two indices obtained in NoGo trials and related to conflict monitoring and response inhibition, respectively (Zhang and Lu, 2012).

The objectives of the present work were (1) to clarify whether fibromyalgia patients show higher interference of attention toward pain stimuli over inhibitory control (using both behavioral and brain activity indices); (2) to assess whether the amplitudes of N2 and P3 to painful faces are related to the core symptoms of fibromyalgia (such as pain severity, catastrophism, depression, or sleep disturbances). Given that previous studies have reported that attention to negative emotions impairs inhibition (Lindström and Bohlin, 2012), we expected to find increased errors and longer reaction times, as well as larger modulation of ERPs and time-frequency indices, in response to pain faces, although those effects should be more pronounced in the patients suffering from FM. We also expected to find a significant correlation between the ERP amplitudes and the severity of the clinical symptoms.

## Materials and Methods

### Participants

A sample of 28 women with FM and 29 healthy women, matched for age, education, laterality, and menopausal status, were enrolled in this study. The inclusion criterion for the FM group was a diagnosis of the disease by a health specialist according to the ACR criteria for fibromyalgia. Exclusion criteria were mental illness or psychiatric disorders (except anxiety and depression). In addition, healthy controls (HCs) should not have any chronic pain condition. For ethical reasons, patients were not asked to withdraw prescribed medical treatments. Demographic characteristics of the groups are shown in Table 1.

**Table 1:** Demographic characteristics, and results of *t*-tests and χ^2^ comparisons between the group of fibromyalgia patients (FM) and healthy controls (HC).

	**FM**	**HC**	***t, χ**^**2**^*	***p***
	***N** = 28*	***N** = 29*		
Age *M (SD)*	50.11 (9.89)	47.83 (11.06)	*t* = 0.756	0.453
Education (%)			χ2 = 0.242	0.886
Primary School	33.3	37.9		
High School	40.7	34.5		
Higher studies	25.9	27.6		
Menopausal women (%)	53.8	46.2	χ2 = 0.617	0.432
Right Handed (%)	96.3	96.6	χ2 = 2.01	0.367

*M (SD), mean (standard deviation)*.

The participants gave their written informed consent for their participation in the study, approved by the Galician Autonomous Committee for Research Ethics (2013/582), and conducted in accordance with the Declaration of Helsinki.

### Measures

Clinical and sociodemographic data on the participants were obtained via a semi-structured interview.

A series of visual-analogical scales (VAS) were created *ad-hoc* to evaluate the main symptoms of FM, as well as the general health condition of the participants. The VAS were administered in paper-and-pencil format and consisted of 10 cm horizontal lines in which the participants had to indicate their condition on the following variables: pain, health status, stiffness, fatigue, mood, headache, and sleep quality. All of them were presented in a manner that the left end indicated the best condition and the right end the worst.

The Spanish version of the *Beck Depression Inventory* (BDI; Beck et al., 1996), validated by Sanz et al. (2003) was used to assess depressive symptoms.

The Spanish version of the *Pittsburgh Sleep Quality Index* (PSQI; Jiménez-Genchi et al., 2008) was self-administered to assess sleep quality and dysfunction during the previous month.

The Spanish version of the *Pain Catastrophizing Scale* was also administered (PCS; Sullivan et al., 1995; García Campayo et al., 2008). In addition to the total score, the 3 factors described in the original study (rumination, magnification, and helplessness) were taken into account.

In the *Perceived Stress Scale* (PSS; Cohen et al., 1994; Remor, 2006), subjects had to indicate which of the several statements best described how they felt during the past week, depending on how unpredictable, uncontrollable, and overloaded respondents find their life. It is considered a measure of the degree to which situations in participants' lives are appraised as stressful.

The Spanish version of the *Memory Failures of Everyday Questionnaire* (Sunderland et al., 1984; Lozoya-Delgado et al., 2012) was also administered. The *MFE-30* is a 5-point Likert (between “never” and “very often”) questionnaire that comprises 30 items related to complaints in different cognitive domains. The *MFE-30* Total score (range 0–120) was calculated as the sum of all items. This score was ranked into four categories: 0–7, 8–35, 36–50, and over 50, indicating optimal performance, normal, mild deterioration, and moderate deterioration, respectively.

The above instruments were used to explore the core clinical symptoms of fibromyalgia, including also variables, such as catastrophism or perceived stress, which may be relevant for the study of attentional bias toward pain-related information. Since inhibition is an executive function, we also explored whether the indices obtained in the Go-NoGo task were related to the subjective cognitive complaints reported by the patients.

The handedness of participants was assessed by administration of the *Edinburgh Handedness Inventory* (Oldfield, 1971).

### Procedure and Stimuli

**Figure 1:**
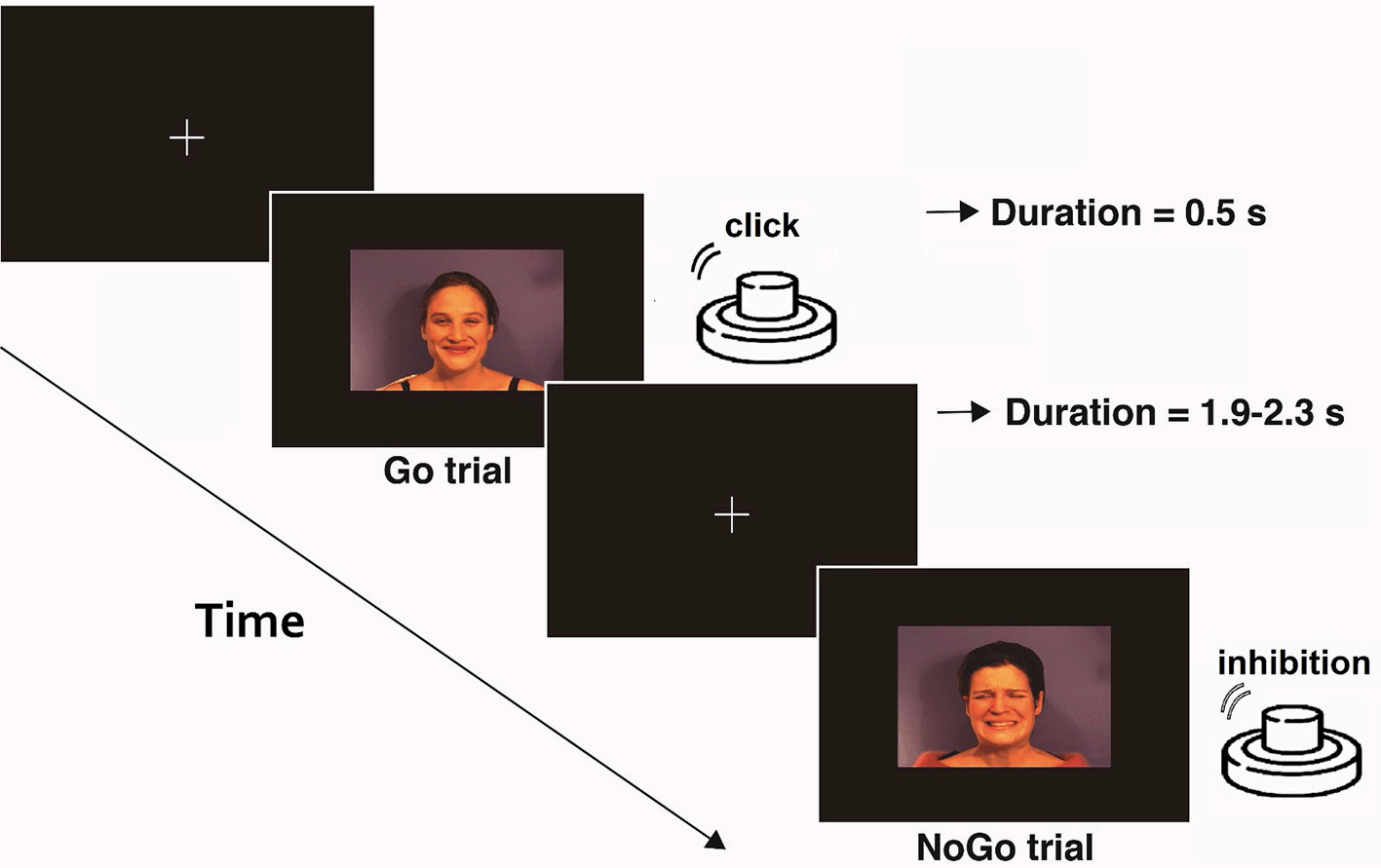
Schematic example of the emotional Go/NoGo task. In each of the three blocks, one type of stimulus (pain, neutral, and happy face) was considered as Go-stimulus. Participants were asked to press the button when a Go stimulus was presented and inhibit that action under the different stimuli (NoGo).

Demographic and clinical information was obtained during the evaluation session, once participants had provided written informed consent to take part in the study. The assessment procedure was administered to both FM patients and HCs. Following the initial interview and the administration of the questionnaires, the EEG recordings took place in a poorly lit room protected from external noise. The Go/NoGo task was designed and presented using Psychopy software (Peirce, 2007) on a 17-inch, 60 Hz LCD monitor at a distance of 80 cm from the subject. The stimuli consisted of micro-films, created and validated by Simon et al. (2008). The original videos had a duration of 1 s, but for our study, we used the last 500 ms of them, when the emotion is well-defined by the actors and is easier to identify (see Figure 1).

The participants were instructed to fix their gaze on a cross at the center of a computer screen during the execution of the task. A total of 240 micro-videos of facial expressions (happy, pain, and neutral) were presented to them in three blocks. In each block, participants were asked to press a button with their index finger button as quickly as possible whenever a specific expression (neutral, happy, or pain) was displayed, and inhibit this response when the expression displayed was different. Each block consisted of 40 Go trials and 40 NoGo trials. The interval between stimuli was 1.9–2.3 s. The order of the blocks was balanced among the participants, who had a resting period between blocks. Prior to registration, 10 practice trials were presented with videos different from those used in the task, to ensure that the instructions were fully understood and that facial expressions were correctly identified.

### EEG Recording and Analysis

To capture the electroencephalographic signal, we used an actiCAP elastic cap with 32 electrodes placed according to the 10–10 International System and a Brain Vision actiCHamp amplifier (Brain Products Inc.). The ground was placed at FPz, and reference at FP1, which was reinstated after re-referencing. One additional electrode was placed 1 cm below the right eye, and vertical ocular movements were monitored by calculating the difference between this electrode and FP2. Electrode impedances were kept below 10 kΩ, and the signal was digitized at 500 Hz with an on-line bandpass filter of 0.01–200 Hz, and a notch filter at 50 Hz.

**Figure 2:**
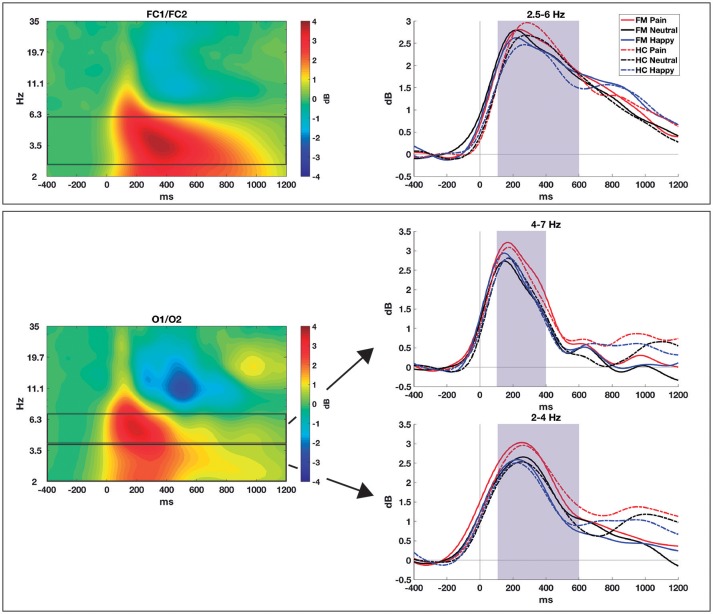
Time-frequency decomposition of the EEG to pain, neutral, and happy faces obtained from patients with FM and healthy controls in NoGo trials. Upper row: mid-frontal theta (FC1/FC2); lower row: posterior alpha and theta (O1/O2). The spectrograms averaged across conditions and groups are shown in the left column, while the right column shows the time course, for each group and condition, in the selected frequency bands. Shaded areas show the time windows selected for statistical comparisons.

EEG data were analyzed using the EEGlab 13.3 toolbox (Delorme and Makeig, 2004). They were re-referenced to an average reference. Data from electrodes with excessive noise were replaced using the Spherical Spline Interpolation method. Nine channels were interpolated for the FM group, and seven for healthy controls. Those segments with large ocular or other artifacts were rejected by visual inspection. Data were down-sampled to 250 Hz and digitally filtered using a 0.5 Hz high-pass FIR filter (order = 3,301) and a 30 Hz low-pass FIR filter (order = 175). Epochs of −800 to 1,800 ms post-stimulus were extracted. An independent component analysis (ICA) was applied to eliminate artifact-related components due to EOG or muscle activity. Linear trends were removed from epochs. Epochs with values exceeding ± 100 μV from −600 to 1,500 ms (excluding the ocular electrodes) were removed. There were no group differences in the number of artifact-related ICAs removed [mean FM = 2.96, *SD* = 1.4; mean HC = 2.77, *SD* = 1.2; *t*_(57)_ = 0.56; *p* = 0.58] (see the final number of epochs included for each group and facial expression in **Table 4**). In addition, to avoid group bias during data pre-processing, the researcher was blind to the group to which each of the EEG recordings belonged. The EEG was re-referenced using the Reference Electrode Standardization Technique (REST) (Yao, 2001), a mathematical procedure that recomputes the reference to a point at infinity. REST re-referencing was computed using a MATLAB toolbox provided by the authors of this method (Dong et al, 2017). The baseline was corrected from −200 to 0 ms. The time-frequency decomposition was performed by calculating the inverse Fast Fourier Transform of the multiplication of the EEG power spectrum by the power spectrum of different complex Morlet wavelets. Wavelets were created in 25 logarithmically-increasing steps (from 2 to 35 Hz), with 3 cycles at the lowest frequency up to 8 at the highest frequency, also in logarithmically-increasing steps. Event-related spectral perturbation was then normalized to decibel (dB) transformation, using the mean power from −400 to −150 ms as the baseline. We analyzed power values of theta over midfrontal areas (FC1 and FC2), the location where these oscillations are frequently observed (Cavanagh and Frank, 2014). Given the visual nature of the stimuli, we also measured delta and theta bands over posterior visual areas (O1 and O2). We first averaged the spectrograms of all conditions in both groups together and then we selected the time-frequency windows where the power modulation was maximum, this window selection method is independent of group/condition differences (Cohen and van Gaal, 2013). The power peak for midfrontal theta was around 3.5 Hz, while posterior theta showed its peak at around 5 Hz. We found an increase in the power of slower oscillations (2–4 Hz) over posterior areas. Given that they can be useful for acquiring insight into the processing of dynamic visual stimuli, we also compared the posterior delta power between groups and conditions (see Figure 2).

We also measured the mean amplitude of P3 at the Cz electrode in a window from 350 to 600 ms, and the mean amplitude of N2 at the Fz electrode in a window from 250 to 340 ms only in NoGo trials. The selection of electrodes was based on previous reports (Falkenstein et al., 1999), while the time windows were selected based on the time when these components showed their greatest amplitude.

### Statistical Analysis

Means and standard deviations were used to describe the quantitative variables, while absolute frequencies and percentages were used for the qualitative measures. Differences between FM and HC groups in clinical and sociodemographic variables were analyzed using Student's *t*-test or Chi-square test, depending on the type of variable considered. Repeated measures ANOVAs with Group (2 levels: FM, HC) as between-subject factor, and Facial Expression (3 levels: happy, pain, neutral) as within-subject factor, were performed for behavioral indices (reaction times and hits), for midfrontal theta, posterior theta, and posterior delta power, and for the amplitude values of P3 and N2. The Bonferroni correction method was applied for *post-hoc* multiple comparisons. Associations between ERPs amplitudes and clinical variables for FM patients were quantified using Pearson correlations. To overcome the problem of multiple comparisons in the correlations, the False Discovery Rate (FDR) correction—using the “fdr” function of EEGlab—was applied.

All statistical analyses were performed using SPSS version 20 (Statistics, 2013).

## Results

### Clinical Characteristics

There were no significant differences between the FM and HC groups in terms of age, education, menopausal status, or laterality (Table 1).

Regarding clinical variables, the comparisons between the FM and HCs were statistically significant for all the scales and subscales analyzed (Table 2). Concerning VAS, the highest scores for the patients' group were on the pain, stiffness, fatigue, and sleep quality scales, the core symptoms of fibromyalgia. With respect to the *Pain Catastrophizing Scale* (PCS), FM patients obtained an average score of 23.72 (*SD* = 15.26) and the control group an average of 11.00 (*SD* = 8.93). It should be noted that the groups differed in all the 3 factors that comprise the scale. As for the *Beck Depression Inventory* (BDI), we also found significantly higher means for the FM group (mean = 20.16) compared to the control group (mean = 10.17). Patients also showed worse sleep quality in the *Pittsburgh Sleep Quality Index* (PSQI) than healthy controls (13.04 vs. 5.75), broader subjective cognitive complaints in the *Memory Failures of Everyday Questionnaire* (MFE-30) (50.95 vs. 26.16) and higher stress levels in the *Perceived Stress Scale* (PSS) compared to the control group (31.78 vs. 21.71).

**Table 2:** Clinical variables of FM patients and healthy controls (HC).

	**FM**	**HC**	***t***	***p***
**VAS** ***M (SD)***				
Pain	6.61 (1.7)	3.12 (3.6)	4.433	** < 0.001**
Health	5.92 (2.2)	3.45 (3.1)	3.271	**0.002**
Stiffness	7.98 (2.1)	2.30 (2.8)	8.122	** < 0.001**
Fatigue	7.48 (1.8)	3.14 (2.5)	6.996	** < 0.001**
Mood	5.45 (3.0)	3.12 (2.4)	2.913	**0.006**
Headache	4.85 (3.1)	1.68 (2.5)	3.966	** < 0.001**
Sleep quality	7.46 (2.6)	3.56 (3.0)	4.822	** < 0.001**
**BDI** ***M (SD)***				
Total score	20.16 (10.4)	10.17 (6.0)	4.029	** < 0.001**
**PSQI** ***M (SD)***				
Total score	13.04 (4.9)	5.75 (3.8)	5.484	** < 0.001**
**PCS** ***M (SD)***				
Rumiation	7.50 (5.2)	4.04 (3.4)	2.826	**0.007**
Magnification	5.00 (3.7)	2.84 (2.4)	2.453	**0.018**
Helplessness	10.92 (7.1)	4.11 (4.3)	4.168	** < 0.001**
Total score	23.72 (15.3)	11 (8.9)	3.648	**0.001**
**PSS** ***M (SD)***				
Total score	31.78 (11.1)	21.71 (8.3)	2.923	**0.006**
**MFE-30** ***M (SD)***				
Total score	50.95 (23.87)	26.16 (13.38)	4.012	**< 0.001**

*FM, fibromyalgia; HC, healthy controls; t, Student's t; M (SD), mean (standard deviation); VAS, Visual Analogue Scale; BDI, Beck Depression Inventory; PSQI, Pittsburgh Sleep Quality Index; PCS, Pain Catastrophizing Scale; PSS, Perceived Stress Scale; MFE-30, Memory Failures of Everyday Questionnaire*.

### Behavioral Data

The behavioral data obtained by FM patients and HCs in the Go and NoGo trials for happy, pain, and neutral stimuli are shown in Table 3.

**Table 3:** Behavioral data (reaction times in milliseconds [RT] and percentage of hits) in the Go and NoGo trials for pain, neutral, and happy facial expressions in fibromyalgia (FM) patients and healthy controls (HC).

	**Pain**		**Neutral**		**Happy**	
	**FM**	**HC**	**FM**	**HC**	**FM**	**HC**
**Go**						
RT	641 (97)	659 (134)	622 (130)	612 (119)	595 (83)	586 (98)
% hits	87.3 (8.4)	82.4 (10.4)	95.2 (7.9)	95.0 (7.8)	96.5 (5.1)	96.9 (4.9)
**NoGo**						
% hits	88.6 (7.3)	90.6 (5.6)	96.7 (4.3)	98.7 (2.8)	93.6 (8.9)	94.1 (5.8)

There was a significant effect of Facial Expression for the reaction times in Go trials [*F*_(2, 114)_ = 18.34; *p* < 0.001; η_*p*_^2^ = 0.243; Pain = 650 ± 118 ms; Neutral = 617 ± 123; Happy = 590 ± 90], as well as for the percentage of correct answers in both the Go [*F*_(2, 114)_ = 46.59; *p* < 0.001; η_*p*_^2^ = 0.450; Pain = 84.7 ± 9.7%; Neutral = 95.1 ± 77.8; Happy = 96.7 ± 4.9] and NoGo [*F*_(2, 114)_ = 40.40; *p* < 0.001; η_*p*_^2^ = 0.415; Pain = 89.7 ± 6.5%; Neutral = 97.7 ± 3.7; Happy = 93.9 ± 7.4] trials. A posteriori contrasts showed that, in the Go trials, faces of pain were associated with slower reaction times than neutral (*p* < 0.001) and happy (*p* < 0.001) faces and, moreover, they were also associated with more errors than neutral (*p* < 0.001) and happy (*p* < 0.001) faces in both groups. Happy faces showed the fastest reaction times and the largest hit percentages. There was no significant interaction between emotion and group. As for the NoGo trials, pain faces were also associated with the worse hit percentages in both groups (*p* < 0.001). In this case, when it comes to inhibiting the response, neutral stimuli were associated with the best performance.

### Electrophysiological Data

For time-frequency data (see Figure 2), we measured midfrontal theta oscillations (2.5–6 Hz) over FC1 and FC2 and from 100 to 600 ms. An effect of Facial Expression was found for theta power [*F*_(2, 114)_ = 6.61; *p* = 0.002; η_*p*_^2^ = 0.104; Pain = 3.06 ± 1.36 dB; Neutral = 2.72 ± 1.20; Happy = 2.63 ± 1.15), with differences between pain and neutral (*p* = 0.005) and pain and happy faces (*p* = 0.017) after *post-hoc* comparisons. No significant Group effect [*F*_(1, 57)_ = 0.05; *p* = 0.94] or interaction [*F*_(2, 114)_ = 2.41; *p* = 0.094] were observed.

Posterior delta activity (2–4 Hz)—measured over O1 and O2 electrodes and from 100 to 600 ms—showed a main effect of Facial expression [*F*_(2, 114)_ = 4.21; *p* = 0.017; η_*p*_^2^ = 0.069; Pain = 2.34 ± 1.42; Neutral = 1.94 ± 1.31; Happy = 1.81 ± 1.55], with significant differences only between pain and neutral conditions (*p* = 0.040) in pairwise comparisons. No Group difference [*F*_(1, 57)_ = 0.03; *p* = 0.85] or interaction [*F*_(2, 114)_ = 0.05; *p* = 0.95] were found significant.

Posterior theta oscillations (4–7 Hz)—measured over O1 and O2 from 100 to 400 ms—also showed an effect of Facial expression [*F*_(2, 114)_ = 4.85; *p* = 0.010; η_*p*_^2^ = 0.08; Pain = 2.99 ± 1.66; Neutral = 2.59 ± 1.70; Happy = 2.59 ± 1.53]. Again, *post-hoc* comparisons showed differences between pain and neutral (*p* = 0.043) and pain and happy conditions (*p* = 0.009), with no significant Group effect [*F*_(1, 57)_ = 0.05; *p* = 0.83] nor Group × Facial expression interaction [*F*_(2, 114)_ = 0.65; *p* = 0.52].

Concerning ERPs, Table 4 shows the average amplitude (μV) for the two ERPs components analyzed.

**Table 4:** Number of EEG epochs and average amplitudes in μV (SD in parentheses) for the P3 and N2 components (NoGo trials) in patients with FM and healthy control (HC) subjects for each type of facial expression.

	**Pain**		**Neutral**		**Happy**	
	**FM**	**HC**	**FM**	**HC**	**FM**	**HC**
Number of EEG epochs	32.5 (2.0)	32.7 (1.5)	34.20 (1.9)	34.5 (1.7)	32.8 (2.5)	32.1 (2.0)
P3 NoGo	3.44 (2.9)	3.37 (3.3)	3.40 (2.4)	2.94 (2.8)	2.60 (2.5)	3.16 (3.0)
N2 NoGo	−4.52 (4.1)	−5.02 (3.2)	−4.40 (3.7)	−4.10 (2.9)	−4.69 (3.8)	−4.18 (2.8)

For P3 amplitude, the results showed a significant effect of Facial Expression [*F*_(2, 114)_ = 3.55; *p* = 0.032; η_*p*_^2^ = 0.059]. Subsequent contrasts showed significantly larger P3 amplitude for pain (mean = 3.41 ± 3.09 μV) than for happy faces (mean = 2.89 ± 2.77 μV) (*p* = 0.027). No significant Group effect was found significant.

**Figure 3:**
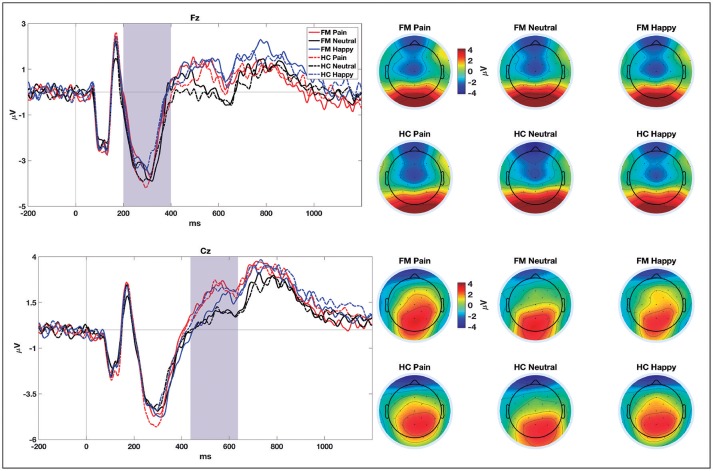
ERPs grand-averages (at Fz and Cz electrodes) for pain, neutral, and happy NoGo trials in patients with fibromyalgia (FM) and healthy controls (HC). Shaded areas show the time windows selected for statistical comparisons. Topographical distribution of brain electrical activity (average of the selected time windows) for each group and condition are shown on the right-hand side.

For N2, the ANOVA showed a significant Facial Expression × Group interaction for the amplitude of this component [*F*_(2, 114)_ = 3.42; *p* = 0.036; η_*p*_^2^ = 0.057]. A posteriori contrasts showed significant differences between pain (mean = −5.02 ± 0.66 μV) and neutral faces (mean = −4.10 ± 5.88 μV), (*p* = 0.001); and between pain and happy faces (mean = −4.18 ± 0.59 μV), (*p* = 0.005), only for the control group. For the patients' group, there was no difference in the amplitude of N2 among any emotional category. The waveforms obtained in the NoGo trials at Fz and Cz electrodes for all the stimuli are shown in Figure 3.

To clarify whether there is a relationship between the amplitude of the N2 and P3 components for pain faces and the clinical variables, Pearson correlations were performed with the FM patients' data. Two of the correlations showed a *p*-value < 0.05: the amplitude of N2 and the quality of sleep measured with the PSQI (*r* = 0.516; *p* = 0.007), and the amplitude of P3 and the level of pain assessed by the VAS (*r* = −0.466; *p* = 0.016). However, none of these 2 correlations survived the FDR correction. No other correlation between ERPs and clinical indices was found significant.

## Discussion

The objective of this study was to test whether patients with fibromyalgia (FM) show a larger interference of attention toward pain-related stimuli over inhibitory control. We recorded brain electrical activity during a Go/NoGo emotional task, in which the participants had to respond or withhold the response to dynamic images of faces with different expressions. Given the dynamic nature of the stimuli, we performed EEG time-frequency analyses (midfrontal theta and posterior delta and theta) and also obtained the ERPs N2 and P3 in NoGo trials.

First, our data support that attention to pain faces interferes with response inhibition. Behaviorally, pain expressions were associated with a higher percentage of errors and slower reaction times. This result suggests that pain-related stimuli are more conflicting and may engage more attentional resources to their processing, resulting in longer reaction times. Nevertheless, this effect was not different in patients and controls.

Pearce and Morley (1989) were pioneers in the study of attentional bias (AB) in chronic pain. Using an emotional Stroop task, they found that FM patients had longer reaction times in naming the color of words related to their pain condition than to those with neutral or emotionally negative semantic content. This was taken as evidence that chronic pain patients show AB toward pain-related information, as revealed by their focus on a dimension irrelevant to the task—facial expression—, causing a slowdown response to the color of the word (Pearce and Morley, 1989). Nevertheless, literature results are far from conclusive.

The study of AB in patients with FM has also yielded contradictory findings. González et al (2010) used an emotional Stroop task and explored the possible mediator role of anxiety in AB. Paradoxically, they found that FM patients were significantly slower in the color denomination of neutral words while showed a non-significant tendency to be slower in symptom-related and negative valence words. These effects were not mediated by anxiety, but by the degree of perceived displeasure associated with negative stimuli. In contrast, Duschek et al. (2014) found a strong emotional interference in FM patients in relation to healthy subjects, manifested as a greater response delay in naming the color of negative words compared to neutral adjectives (Duschek et al., 2014). They suggested that, in FM patients, negative information recruits a disproportionate amount of cognitive resources, thereby slowing concurrent processes.

Although we used a different strategy—to analyze the influence of attention to pain over inhibitory control—our behavioral results are in line with the conclusions of the meta-analysis conducted by Crombez et al. (2013) that the AB toward negative emotional information is similar in chronic pain and healthy subjects. Also, behavioral data agree with the results of Glass et al. (2011), who observed the same RTs and accuracy in FM patients during a simple Go/NoGo task. Since previous studies have suggested greater effectiveness of images to represent emotions—as compared to words—(Crombez et al., 2000, 2013; Van Damme et al., 2010), in this paper we used images and, to make them more realistic and with a greater impact, we presented them dynamically. Despite this choice, we have not been able to observe a pattern of response that confirms larger interference of pain stimuli over inhibitory control in patients than in healthy controls.

To better know the temporal characteristics of neural activation involved in response inhibition, we recorded EEG during the performance of the task and analyzed it with two different methodologies. Since the ERPs components may be reflecting overlapping theta and delta activity (Harper et al., 2014), and given the dynamic nature of our stimuli, we applied time-frequency decomposition to the EEG. We analyzed midfrontal theta, and posterior delta and theta bands, and found that all of them were significantly larger for pain faces, but no between-group differences emerged. Concerning ERPs, and also in parallel with the time-frequency and behavioral results, NoGo-P3 was larger when pain faces were presented, but the effect was similar for the patients and healthy controls. In this vein, our results also contrast with previous studies. In 2013, Mercado and colleagues using a Stroop emotional paradigm found larger amplitudes of the P450 component in the patients when processing symptom-related stimuli compared to the rest of the stimuli. This was interpreted as an increase in prefrontal neuronal activity when processing information related to FM symptoms. As they did not find between-groups differences in behavioral outcomes, they hypothesized that an increased P450 component could be reflecting the activation of a compensation mechanism to keep cognitive task performance through additional cognitive inhibitory resources (Mercado et al., 2013). González-Roldán et al. (2013) also found that FM patients had larger N100 amplitudes to facial expressions of pain and anger than happiness. Unlike previous studies, which used pure and simple attentional tasks, we used a more demanding paradigm where subjects had to identify the facial expression and then prepare or withhold their motor response. Even so, and using stimuli directly related to FM symptoms such as pain, we were not able to identify differences in brain activity related to response inhibition. The fact that time-frequency analyses and P3 data are fully consistent adds robustness to the main finding of this paper, which does not confirm that patients have difficulty disengaging their attention from pain-related information while performing an inhibition task.

Our results are in line with a recent study by Sitges et al. (2018) who compared FM patients with pain-free subjects in a Go/NoGo task using pain, happy and neutral faces and did not find group differences in N200 and P300 amplitudes, what was interpreted as a lack of significant impairment in response inhibition due to pain. Nevertheless, they contradict Glass et al. (2011) report on decreased activation in cortical structures of the inhibition network in FM patients, which was interpreted by the authors as a consequence of neural resources being used for pain processing.

The only significant electrophysiological difference between the groups is related to the amplitude of N2, for which we found an interaction of group with the type of facial expression. Only for healthy participants, N2 showed differences between pain and both neutral and happy faces. The N2 component obtained in the NoGo trials has been linked to inhibitory processes, and more recently, to conflict detection (Falkenstein et al., 1999; Donkers and Van Boxtel, 2004). Therefore, the data seem to indicate that, in healthy controls, the processing of facial expressions of pain may produce more conflict; whereas in patients, who are more familiarized to pain and may require fewer attentional resources to identify it, the conflict would be lesser. This would explain the lack of N2 amplitude modulation in the presence of pain faces in the patients.

Alternatively, this result could also indicate a delay in emotional processing in the patients, as about 300 ms post-stimulus they have not yet differentiated the emotional content. However, the lack of a significant difference in reaction times between patients and healthy controls does not seem to support this explanation. Finally, it could also be interpreted as less emotional reactivity in patients with fibromyalgia. In this regard, there are studies that corroborate that alexithymia, a personality construct characterized by difficulty in identifying and communicating feelings (Taylor, 1984), may play a role in the maintenance or exacerbation of fibromyalgia symptoms (van Middendorp et al., 2008; Huber et al., 2009).

To elucidate the relationship between the ERPs obtained in the presence of painful faces and clinical variables, we performed a correlational analysis for the FM group. Although a priori the data suggested two significant correlations, these did not survive the FDR correction. Contrary to what might be expected, we found no correlation between variables such as catastrophism or depression and the ERPs. In this vein, Sitges et al. (2018) did not find significant differences between FM patients with low or high depression on N2 and P3 amplitudes.

Our study adds to a growing body of research that has failed to find evidence of an attentional bias toward pain-related stimuli in patients with fibromyalgia other than the present in healthy subjects. More sensitive paradigms may be necessary to elicit the response bias toward pain stimuli. Also, and despite using dynamic images instead of written words, our stimuli may not have had enough impact, and might not have interfered with emotional processing. Perhaps the impact would have been greater if the images referred to pain experienced by the subject himself, and not to the pain of others since some authors suggest that biases in information processing are increasing and may even be dependent on self-referential coding (Pincus et al., 1998). However, research on pain empathy seems to support the ecological validity and impact of the stimuli used: subjective pain perception has been shown to be enhanced by the observation of other persons in pain (Godinho et al., 2008), and recent imaging studies have shown overlapping activation patterns when subjects feel their own emotions and observe the same emotions in others (Ochsner and Gross, 2008).

Attention influences subjective pain (Miron et al., 1989) and is accompanied by activity changes in pain-related brain areas (Godinho et al., 2008). It is important for future research to fully explore the role attentional bias plays in the causation and maintenance of chronic pain diseases like fibromyalgia, and the potential consequences AB may have upon the patients' quality of life (Schoth et al., 2012). AB to pain-related information may initially be adaptive, allowing us to escape or avoid pain. However, if an attentional bias persists even when pain is inevitable—as in the case of chronic pain—it can only exacerbate pain, disability, and distress (Van Ryckeghem et al., 2013), and enhance symptoms including dyscognition, anxiety, and depression. Hence, AB remains a potentially important factor in the development of chronic pain and fibromyalgia, and a relevant area of focus when developing treatment strategies.

As limitations of the present study, we may underscore the fact that we used equiprobable Go and NoGo trials. Although this is rather frequent in the literature, Wessel (2018) found that slow-paced and equiprobable Go/NoGo tasks were associated with a reduction in frontocentral P3 amplitude. Thus, our task might have not been enough sensitive to capture differences between groups. Although conducting ERP analyses with dynamic stimuli might be questionable, other ERPs studies have also used dynamic facial expressions and found that they even produced greater amplitudes than the static ones (Recio et al., 2011). Another limitation is related to the medication intake monitoring. Due to the slight efficacy of pharmacological treatment, FM patients tend to be polymedicated and subjected to continuous drugs modifications. Participants were asked not to take more medicines than necessary but, for ethical reasons, the prescribed treatment was not withdrawn. Finally, and considering the small effect sizes found, we must bear in mind that “no evidence of effect” does not imply “evidence of no effect.” Thus, larger sample sizes are needed to discard that patients with chronic pain do not exhibit attentional bias.

## Author Contributions

MP-M, AG-V, and MC contributed to the study concept and design and wrote the paper. MP-M and AG-V performed the patient's assessment and EEG recordings. All authors approved the final version of the manuscript for submission.

### Conflict of Interest Statement

The authors declare that the research was conducted in the absence of any commercial or financial relationships that could be construed as a potential conflict of interest.
